# Supporting African communities to increase resilience and mental health of kids with developmental disabilities and their caregivers using the World Health Organization’s Caregiver Skills Training Programme (SPARK trial): study protocol for a cluster randomised clinical controlled trial

**DOI:** 10.1186/s13063-024-08488-w

**Published:** 2024-10-24

**Authors:** Melissa Washington-Nortey, Vibian Angwenyi, Mekdes Demissie, Eva Mwangome, Tigist Eshetu, Hanna Negussie, Kimberley Goldsmith, Andrew Healey, Merga Feyasa, Girmay Medhin, Amanuel Belay, Temesgen Azmeraw, Medhanit Getachew, Rahel Birhane, Carophine Nasambu, Tsegereda Haile Kifle, Angela Kairu, Beatrice Mkubwa, Fikirte Girma, Rehana Abdurahman, Ruth Tsigebrhan, Liya Tesfaye, Leonard Mbonani, Nadine Seward, Tony Charman, Andrew Pickles, Erica Salomone, Chiara Servili, Edwine Barasa, Charles R. Newton, Charlotte Hanlon, Amina Abubakar, Rosa A. Hoekstra

**Affiliations:** 1 Department of Psychology, Institute of Psychology, Psychiatry and Neuroscience (IoPPN), King’s College London, London, UK; 2 Institute for Human Development, Aga Khan University, P.O. Box 30270-00100, Nairobi, Kenya; 3 Centre for Innovative Drug Development and Therapeutic Trials for Africa, College of Health Sciences, Addis Ababa University, Addis Ababa, Ethiopia; 4 Neuroscience Group KEMRI-Wellcome Trust Research Programme, Centre for Geographic Medicine Research (Coast), P.O. Box 230-80108, Kilifi, Kenya; 5 Department of Biostatistics and Health Informatics, Institute of Psychology, Psychiatry and Neuroscience (IoPPN), King’s College London, London, UK; 6 Health Service and Population Research, Institute of Psychology, Psychiatry and Neuroscience (IoPPN), King’s College London, London, UK; 7 Yekatit 12 Hospital Medical College, Addis Ababa, Ethiopia; 8 Nia Foundation, Joy Center for Autism, Addis Ababa, Ethiopia; 9 Kuhenza for Children’s Foundation, Kilifi, Kenya; 10 School of Health and Social Science, University of Edinburgh, Edinburgh, UK; 11 University of Milano-Bicocca, Milan, Italy; 12 Department of Mental Health and Substance Abuse, World Health Organization, Geneva, Switzerland; 13 Department of Psychiatry, University of Oxford, Oxford, OX3 7FZ UK; 14 Department of Psychiatry, WHO Collaborating Centre in Mental Health Research and Capacity-Building, School of Medicine, College of Health Sciences, Addis Ababa University, Addis Ababa, Ethiopia; 15 Division of Psychiatry, Centre for Clinical Brain Sciences, University of Edinburgh, Edinburgh, UK

**Keywords:** Children, Developmental disabilities, World Health Organization, Caregiver Skills Training, Cluster randomised controlled trial, Ethiopia, Kenya, Caregivers, Mental health

## Abstract

**Background:**

Most children with developmental disabilities (DD) live in low- and middle-income countries, but access to services is limited, impacting their ability to thrive. Pilot study findings of the World Health Organization’s Caregiver Skills Training (WHO CST) intervention, which equips caregivers with strategies to facilitate learning and adaptive behaviours in children with DD, are promising but evidence from an appropriately powered trial delivered by non-specialist facilitators is lacking. This study will investigate the effectiveness and the resource impacts and costs and consequences of the WHO CST intervention in four sites in rural and urban Kenya and Ethiopia.

**Methods:**

This is a 2-arm multi-site hybrid type-1 effectiveness implementation cluster randomised controlled superiority trial. After baseline assessments (T0) are completed by participants in clusters comprising 7 to 10 caregiver-child dyads, the clusters will be randomised to either the WHO CST intervention arm or a waitlist enhanced care as usual control arm. Further assessments will be completed at endpoint (T1, 18 ± 2 weeks after randomisation) and follow-up (T2, 44 ± 2 weeks after randomisation). The intervention comprises three individualised home visits and nine group sessions with trained non-specialist facilitators. Participants in the control arm will receive the intervention after completing follow-up assessments. We aim to recruit 544 child-caregiver dyads, evenly distributed across the two arms and countries. The co-primary outcomes are the child-focused Child Behavior Checklist (assessing emotional and behavioural problems) and the caregiver-focused Pediatric Quality of Life Inventory (assessing caregiver quality of life), both assessed at endpoint. Secondary outcome measures comprise the two co-primary outcomes at follow-up and ten additional outcome measures at endpoint, assessing stigma-based experiences, depressive symptoms, household food insecurity, child disciplinary strategies and beliefs, CST knowledge and skill competencies, caregiver and child quality of life, social support, and children’s communication modes and functions. After quantitative follow-up assessments are completed, a mixed-methods evaluation approach will be used to investigate implementation processes and acceptability, feasibility, and potential sustainability of the intervention.

**Discussion:**

The study’s findings will provide evidence of the effectiveness and resource impacts and costs and consequences of a non-specialist-delivered intervention in under-resourced contexts in one low-income and one middle-income country in East Africa. Findings will inform future research, intervention, and policy efforts to support children with DD and their families in under-resourced majority world contexts.

**Trial registration:**

Pan African Clinical Trial Registry PACTR202310908063134. Registered on October 16, 2023.

**Supplementary Information:**

The online version contains supplementary material available at 10.1186/s13063-024-08488-w.

## Administrative information

**Table:** 

Title {1}	Supporting African communities to increase resilience and mental health of kids with developmental disabilities and their caregivers using the World Health Organization’s Caregiver Skills Training Intervention (SPARK trial): study protocol for a cluster randomised clinical controlled trial
Trial registration {2a and 2b}	Pan African Clinical Trial Registry ID#: PACTR202310908063134
Protocol version {3}	5th March 2024 Version 5
Funding {4}	National Institute of Health and Care Research (NIHR—NIHR200842)
Author details {5a}	1. Department of Psychology, Institute of Psychology, Psychiatry and Neuroscience (IoPPN), King’s College London, London, UK 2. Institute for Human Development, Aga Khan University, Nairobi P.O. Box 30,270–00100, Kenya 3. Centre for Innovative Drug Development and Therapeutic Trials for Africa, College of Health Sciences, Addis Ababa University, Addis Ababa, Ethiopia 4. Neuroscience group KEMRI-Wellcome Trust Research Programme, Centre for Geographic Medicine Research (Coast), Kilifi P.O. Box 230–80108, Kenya 5. Department of Biostatistics and Health Informatics, Institute of Psychology, Psychiatry and Neuroscience (IoPPN), King’s College London, London, UK 6. Health Service and Population Research, Institute of Psychology, Psychiatry and Neuroscience (IoPPN), King’s College London, London, UK 7. Yekatit 12 Hospital Medical College, Addis Ababa, Ethiopia 8. Nia Foundation, Joy Center for Autism 9. Kuhenza for Children’s Foundation, Kilifi, Kenya 10. University of Edinburgh, School of Health and Social Science, Edinburgh, UK 11. University of Milano-Bicocca, Milan, Italy 12. Department of Mental Health and Substance Abuse, World Health Organization, Geneva, Switzerland 13. Department of Psychiatry, University of Oxford, Oxford OX3 7FZ, UK 14. Department of Psychiatry, WHO Collaborating Centre in Mental Health Research and Capacity-Building, School of Medicine, College of Health Sciences, Addis Ababa University, Addis Ababa, Ethiopia 15. Division of Psychiatry, Centre for Clinical Brain Sciences, University of Edinburgh, Edinburgh, UK
Name and contact information for the trial sponsor {5b}	Research Governance Office King’s College London 5–11 Lavington Street (3rd floor) London SE1 0NZ rgo@kcl.ac.uk
Role of sponsor {5c}	The sponsor and the funder had no role in the study design, data collection, analysis, interpretation, or writing of the manuscript.

## Introduction

### Background and rationale {6a}

According to global reports, approximately 317 million children and adolescents are at risk of developmental disabilities (DDs) [1]. About 95% of children with DDs, including autism and intellectual disability, live in low- and middle-income countries (LMICs) [2]. Families of children with DDs living in low-resource contexts in Africa experience severe challenges that impact their mental health and quality of life. DDs may be attributed to supernatural causes or seen as a curse or punishment for sin. Consequently, children with DDs and their families are frequently stigmatised, discriminated against and hidden at home, and are often excluded from schools and from participating in community social activities [3, 4]. In addition, limited formal resources exist to care for and support the children and their families in these contexts. Specialists skilled in identifying and managing these conditions are few and often relegated to more affluent, mostly urban areas [5, 6]. As a result, children with DDs are rarely identified and have limited access to formal and informal support outside their families.

To address these gaps in service provision, the World Health Organization (WHO) developed the Caregiver Skills Training (CST) package for families of children with DDs. The CST intervention seeks to improve the outcomes of 2- to 9-year-old children with DDs by teaching their caregivers strategies to promote communication and play, increase adaptive skills, and reduce challenging behaviours [7, 8]. It also seeks to improve caregiver well-being by reducing caregiving stress, promoting caregivers’ self-care practices, and increasing informal engagement among caregivers facing similar challenges [9]. A field-testing initiative of the pre-publication draft of the CST package involving more than 30 countries [7] informed the finalisation of WHO CST materials, which comprise facilitator guides, participant booklets, and an adaptation guide [9]. To date, several studies have investigated the intervention’s feasibility, acceptability, and preliminary effectiveness in improving child and caregiver outcomes with mixed but largely positive results [7, 8]. Our team adapted and pilot-tested the CST intervention in separate studies in Ethiopia and Kenya. In qualitative investigations of the intervention in urban [10] and rural [11] Ethiopia, caregivers perceived the intervention as highly acceptable, noting improvements in knowledge about their children’s conditions, increased confidence in their children’s capacity for learning, and improvements in their own capacity to support and provide care [10]. Caregivers reflected on their child’s skills development and linked the skills gained to strategies they learnt in the CST intervention [10, 11]. Caregivers also reported that participating in the intervention helped to reduce stress and social isolation and increase well-being derived from engaging with others facing similar challenges [10]. A model of CST delivery by non-specialist facilitators under specialist supervision was tested in rural Ethiopia and found to be feasible and acceptable [11]. The CST was similarly acceptable and feasible in Kenya when implemented in informal urban settings and rural areas. Community representatives felt the intervention would help address misconceptions and challenges related to stigma and discrimination frequently experienced by these children and their families. Retention rates were high and caregivers were optimistic that practising the CST strategies would help their children achieve important developmental milestones [12].

Feasibility and acceptability studies from other countries, including Hong Kong, India, Italy, Serbia, and Taiwan, also suggest the CST, including the associated home visits, is acceptable with low attrition rates and positive caregiver reports about the intervention’s comprehensibility, relevance, usefulness, and alignment with personal and family values [13–16]. However, in Hong Kong, researchers noted that although most caregivers felt the intervention was useful, some questioned its unique value compared to other interventions already available to families in that country. Further, others shared reservations about Hong Kong caregivers’ willingness to permit intervention delivery by non-specialist facilitators considering residents’ strong preference for specialist-delivered interventions [16]. These caregiver reservations allude to the resource-rich nature of the implementation context in Hong Kong, which contrasts with the reality in LMICs. Some studies highlighted specific components of the intervention that were considered less acceptable by caregivers. For instance, in Italy, caregivers’ least preferred component of the intervention was the paired practice or role play with other caregivers [15]. In Ethiopia, CST facilitators raised some concerns that caregiver-child play-interaction components of the intervention are unfamiliar to many caregivers [11].

Most quantitative studies so far have been small pre-post pilot investigations without a comparison arm. A pilot in India including caregivers of children with social-communication delays, most of whom had a formal autism diagnosis, suggested participating in the CST intervention may be linked to reductions in caregiver’s stress, increases in their knowledge and skills, and improvements in their children’s communication, social interaction, and adaptive behaviours [17]. In another pre-post intervention study among caregivers of children with DDs in Serbia, the intervention was linked to improvements in children’s speech and language communication and health and behaviour [13]. Further, in Hong Kong and Taiwan, participation in the CST was associated with reductions in children’s autistic symptoms [14] and challenging behaviours [16] and improvements in caregivers’ self-efficacy, emotion regulation, self-care competencies, confidence in the CST strategies, and the management of challenging child behaviours [14, 16] when pre- and post-intervention data were compared.

One of the few studies conducting a randomised controlled trial design was a Pakistan-based effectiveness evaluation of a heavily adapted version of the CST intervention based on a preliminary field-testing draft. It excluded all individualised components and was delivered with other components of the WHO’s Mental Health Gap Action Programme (mhGAP) through a tablet application. Five hundred and forty parents of 2–12-year-old children with DDs participated in the intervention, delivered by trained family volunteers. Its results were less positive: compared to the control arm, caregivers in the intervention arm reported greater health-related quality of life, but no significant differences between arms were found on the primary outcome measure, child functioning, or any other secondary outcome measure examined [18]. The authors attributed the non-significant findings to the heterogeneity of the sample and the relatively short intervention timeframe, which was about 3 weeks shorter than originally planned. Additionally, the substantial adaptations to the intervention itself, which drastically simplified its contents to improve contextual fit and potential scalability, may also have contributed to the non-significant findings.

In contrast, our team in Kenya conducted a pilot study including a control group and found the CST to be effective at reducing children’s internalising and externalising challenging behaviours and caregivers’ stress and depressive symptoms in the intervention arm compared to the control arm [12]. There were also significant improvements in caregivers’ quality of life, favouring the intervention arm.

Additionally, in a randomised controlled trial from Italy of CST against enhanced treatment-as-usual, Salomone and colleagues [19] found that participating in the CST intervention delivered by specialist facilitators in public health settings was associated with significant improvements in the masked-rated primary outcomes assessing caregivers’ skills in supporting joint engagement with their child, and the dyadic fluency of the caregiver-child interaction, compared to caregivers in an enhanced treatment-as-usual control arm, who received a single psycho-education session. Significant treatment effects were also reported for caregiver self-efficacy, stress, and child gestures. A sequential mediation analysis testing the theory of change indicated that changes in caregiver’s skills significantly mediated the effect of treatment at the 3-month follow-up on the dyad primary outcome and the other child outcomes [20].

In summary, preliminary findings from pilot studies and RCTs of the CST (or versions thereof) have been largely promising despite some non-significant findings from specific studies. However, many of these studies have been limited by factors such as small sample sizes [13, 17, 21], a narrow focus on children with autism instead of children with a wider range of DDs [18], and limited implementation in LMIC contexts, where most children with DDs reside. Moreover, many studies evaluated the intervention using specialist-based intervention delivery instead of delivery by non-specialists [17, 21, 22]. The effectiveness of the revised CST has not yet been fully examined among caregivers of children with a wider range of DDs where a task-sharing model employing non-specialist facilitators has been adopted. This protocol paper outlines plans for conducting a 2-arm assessor masked hybrid type-1 effectiveness implementation parallel cluster randomised controlled superiority trial (RCT) to test the effectiveness and the resource impacts and costs and consequences of the CST in four sites in urban and rural Ethiopia and Kenya. We use a cluster design because the intervention is delivered to groups of caregivers.

### Objectives {7}

The primary objective of the trial is to assess the effectiveness of the WHO CST compared to a waitlist enhanced care as usual arm at reducing emotional and behavioural problems in children with DDs and improving their caregivers’ quality of life at endpoint (T1, 18 ± 2 weeks post-randomisation) at the individual level. The secondary objectives are to investigate the effectiveness of the WHO CST intervention compared to the waitlist enhanced care as usual at (i) reducing emotional and behavioural problems in children with DDs at follow-up (T2, 44 ± 2 weeks after randomisation) and (ii) increasing quality of life in caregivers at follow-up; (iii) reducing at endpoint (T1 18 ± 2 weeks post-randomisation) (a) caregivers’ experienced stigma; (b) affiliate stigma; (c) caregivers’ depressive symptoms; (d) household food insecurity; (e) child physical punishment; and increasing at endpoint (f) caregivers’ self-reported skills relating to CST; (g) caregiver quality of life; (h) child quality of life; (i) caregiver social support; and (j) child mode and functions of communication. The main secondary outcome measures are the primary outcomes at follow-up (T2).

We will evaluate the resource impacts and costs and consequences of the WHO CST intervention compared to the waitlist enhanced care as usual. Whether the WHO CST has differing impacts by levels of important baseline variables will be examined through a set of pre-specified exploratory moderation analyses. Additional investigations to assess the impact of contextual factors on implementation success and mediation analyses to determine the extent to which any intervention-induced reduction in child behavioural problems brings about improvement in caregiver quality of life are outlined in the main protocol but will not be reported in the main trial paper.

### Trial design {8}

**Fig. 1:**
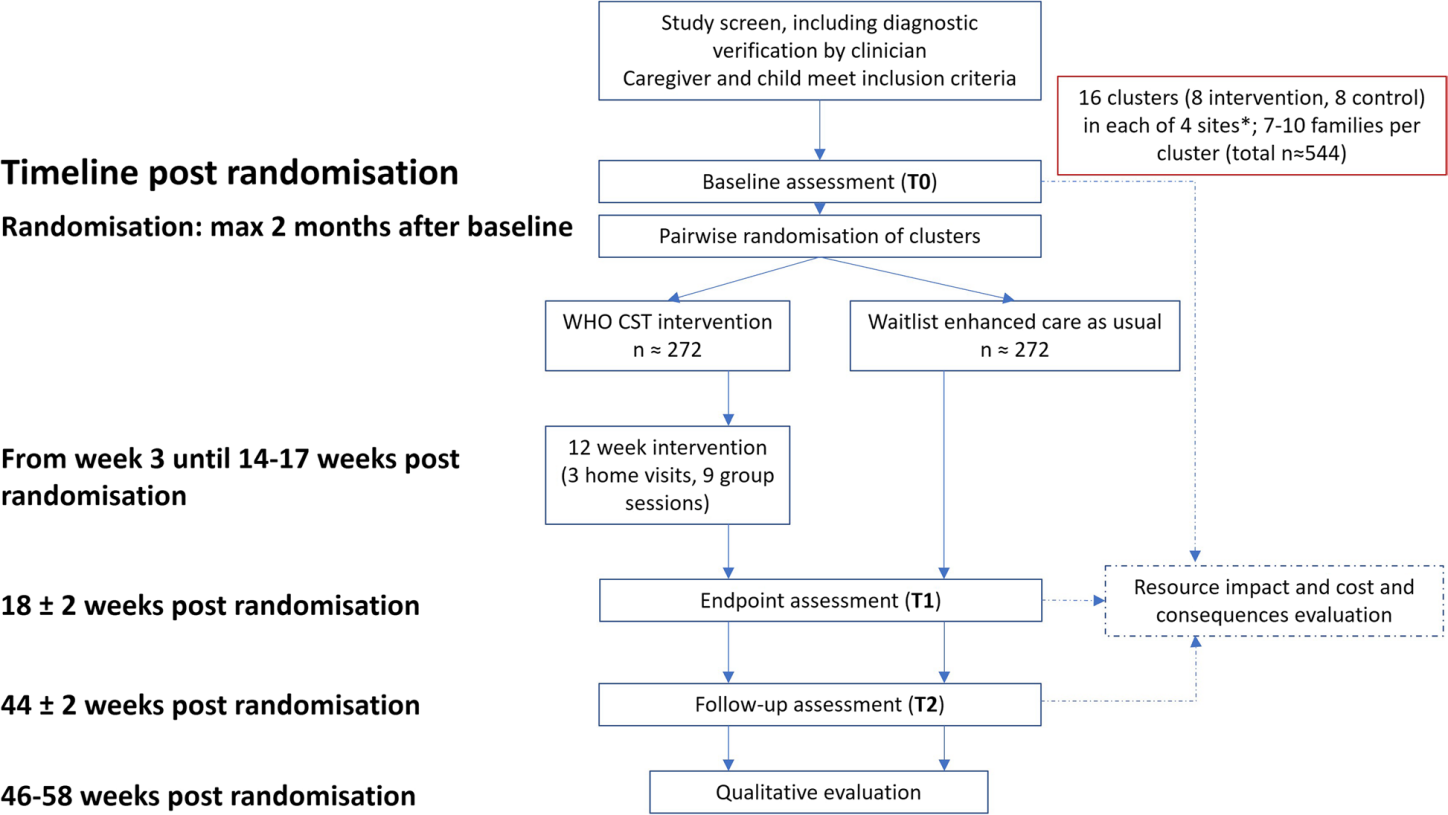
Trial design. Note: *Due to security concerns in the Gurage/Silte site there is a probability that the Ethiopian component of the SPARK trial will be conducted exclusively in Addis Ababa. This site will then comprise 32 clusters (16 intervention, 16 control)

The trial is a 2-arm multi-site hybrid type-1 effectiveness implementation parallel cluster randomised controlled superiority trial with 1:1 allocation ratio (see also Fig. 1).

## Methods: participants, interventions, and outcomes

### Study setting {9}

The study is based in Ethiopia and Kenya, with rural and urban sites in each country to represent a range of contexts comprising different ethnicities, languages, and socio-economic contexts. Each site includes 16 geographic clusters. In Ethiopia, Addis Ababa, the capital city, serves as the urban site, whereas parts of the Gurage and Silte zones form the intended rural site. However, due to on-going security challenges in the Gurage and Silte zones at the time of writing limiting trial implementation, in Ethiopia the trial may be conducted exclusively in Addis Ababa, with a larger number of geographic clusters in this site. Informal settlements in Dagoretti and Ruaraka sub-counties in Nairobi form the urban Kenyan site; Kilifi County serves as the Kenyan rural site. Each cluster represents a geographical area with at least 1000 2–9-year-old children. The unit of clustering in Ethiopia is the catchment area around health centres; catchment areas around selected community health units delineate the clusters in Nairobi. In Kilifi, clusters are formed by a combination of enumeration zones from the Kilifi Health and Demographic Surveillance System, delineating areas with at least 1000 children aged 2–9 years.

### Eligibility criteria {10}

The inclusion criteria for children and caregivers are listed below.

Children:

- Aged between 2 and 9 years, as this is the target age for which the WHO CST intervention was developed. The CST is unsuitable for children under 2 years as it presumes a higher level of developmental maturation and is unlikely to meet the needs of older children because puberty and sexual maturation then become important issues, which are not addressed by WHO CST.
- A DSM-5 diagnosis of one or more specific neurodevelopmental disorders associated with impairments in social and social communication domains, including (1) autism spectrum disorder, (2) intellectual disabilities (intellectual disability; global developmental delay; unspecified intellectual disability), including children with conditions known to cause intellectual disabilities, e.g. Down’s syndrome, Prader-Willi syndrome, and foetal alcohol syndrome, (3) communication disorders: language disorder and social communication disorder only (but not including speech sound disorder, childhood-onset fluency disorder, or unspecified communication disorder).

Caregivers:

- Long-term caring responsibility for a child aged 2–9 years with a DD, preferably as the primary caregiver.
- Resident in the same household as the child and with sufficient contact time during the week with the child with DD (seeing the child at least 5 days a week on average) to carry out homework exercises.
- Able to attend three individual home-based sessions and nine group sessions.
- Intending to stay within the study area for at least 12 months.
- Able to speak Amharic/Kiswahili/Kigiriama/English (as appropriate for the site).
- 18 years of age or older, or an emancipated adult (i.e. younger than 18 years and the biological parent of the child with DD).

The exclusion criteria comprise the following.

Children:

- Having another neurodevelopmental disorder (ADHD, motor disorders, specific learning disorder) without the presence of autism, intellectual disability or global developmental delay, or communication disorder. The CST and its strategies focus on addressing delays and impairments in the social and communication domain; children with diagnoses in these other categories of neurodevelopmental disorders have needs that are not well aligned with the strategies offered in the intervention.
- Epilepsy only (with no co-occurring neurodevelopmental disorder).
- In need of urgent medical attention for other conditions.
- Found to be severely malnourished: assessed using Mid Upper Arm Circumference, with < 115 mm as cut-off following WHO guidance (for children up to 5 years) or using body mass index (for children over 5 years) with the specialist confirming malnutrition is so severe that the child is unlikely to directly benefit from the CST intervention.
- Having co-occurring physical or sensory disabilities or health problems that mean the strategies taught in the CST are unlikely to benefit the child, including:
  o Severe to profound hearing loss, severe visual impairment, or totally blind (children with mild/moderate impairments are eligible for inclusion).
  o Severe motor impairment: inability to sit independently; inability to move upper extremities independently.

Caregivers:

- Living outside the delineated study cluster.
- Previously participated in the CST intervention in Ethiopia or Kenya.
- In need of urgent medical attention.
- Having sensory disabilities or intellectual disabilities that severely limit their ability to participate in CST group sessions and/or to implement CST strategies with their child, including (1) severe to profound hearing loss, severe visual impairment, or totally blind (caregivers with mild/moderate impairments are eligible for inclusion), (2) speech impairment-related communication difficulties, and (3) moderate, severe, or profound intellectual disability.

### Who will take informed consent? {26a}

An independent research assistant employed by the project will obtain informed consent from eligible caregivers after completing eligibility checks and before randomisation. We will not take assent from children as they are young (under 10 years of age) and DDs impact their ability to provide assent. The research assistant will explain the study procedures and issues related to voluntary participation, confidentiality, withdrawal, and data management in the language most familiar to the caregiver (Kigiriama, Kiswahili, English, or Amharic) using the information sheets linked to the consent forms. They will invite and respond to the caregiver’s questions. Caregivers will also be allowed to consult with others including family members, if desired. They will be given time to reflect on whether they wish to participate in the study: 30 min if they want to consent on the same day and at least 24 h if they desire to consent later. Only the main caregiver participating in the CST and completing the associated research assessments will be required to consent. Literate caregivers will sign a copy of the consent form, and non-literate caregivers will provide a fingerprint stamp after a literate, independent witness has confirmed the information sheet. All caregivers will be given a copy of the information sheet and consent form to take home.

### Additional consent provisions for collection and use of participant data and biological specimens {26b}

Not applicable, we will not be collecting any biological specimens.

## Interventions

### Explanation for the choice of comparators {6b}

Eligible participants will be assigned to the intervention arm or a waitlist enhanced care as usual control arm at the cluster level. In all SPARK clusters, healthcare workers in the local health centres (Ethiopia) and health or education workers in local health centres and/or local Education Assessment and Resource Centres (Kenya) will receive training based on principles outlined in the developmental disorders module of the WHO’s mhGAP intervention guide [8]. The module provides guidance on investigating, assessing, and managing child and adolescent DDs. Very few health workers in Kenya and Ethiopia are trained in the identification of and care for children with DDs. Therefore, ensuring access to health or education workers who have received this training constitutes enhanced care as usual. The control arm will wait and receive the WHO CST intervention after the T2 follow-up data collection is completed.

### Intervention description {11a}

The CST intervention teaches strategies to caregivers of 2–9-year-old children with DD to promote learning, communication, engagement, and adaptive behaviours and reduce challenging behaviours in their children [9]. It also seeks to improve caregiver well-being by reducing caregiver stress, promoting caregivers’ self-care practices, and increasing informal engagement among caregivers facing similar challenges. The CST intervention comprises nine group sessions and three individualised home visits. The group sessions focus on getting and keeping children engaged (sessions 1 and 2); building home and play routines (session 3); understanding and promoting communication (sessions 4 and 5); teaching daily living skills (session 6); preventing and responding to challenging behaviours (sessions 7 and 8); and problem solving and self-care (session 9). Home visits focus on defining family-specific goals and identifying and addressing additional support needs (home visit 1), coaching, evaluating progress, troubleshooting, and supporting independent practice (home visits 2 and 3). The intervention is delivered weekly by trained non-specialist facilitators under the supervision of specialist professionals (i.e. master trainers and supervisors). Facilitator guides and participant booklets, developed by the WHO team and adapted and translated into the local languages by our research team, will be used to deliver the group sessions and home visits. There will be one intervention group per cluster for clusters that are randomised to the intervention arm.

### Criteria for discontinuing or modifying allocated interventions {11b}

No discontinuation criteria or plans to modify allocated interventions have been outlined for this trial. However, should the Data Safety and Monitoring Board (DSMB) determine the risks of participation outweigh the benefits for specific or all participants, they can recommend termination of the trial. The Trial Steering Committee will consider the advice of the DSMB and will vote to make a final decision on whether these recommendations should be implemented.

### Strategies to improve adherence to interventions {11c}

Prior to initiating the trial, our adherence strategies include (1) providing extensive theoretical and practical training to master trainers, supervisors, and non-specialist facilitators and (2) assessing their fidelity levels with the Activity Completion Checklists (ACC) and competency levels with the WHO CST Adult–child Interaction Fidelity rating scale and the ENhancing Assessment of Common Therapeutic factors (ENACT) [23]. During the trial, we will maintain intervention adherence in the group sessions by ensuring continued supervision of non-specialist facilitators by CST supervisors. CST supervisors are specialists (in psychiatry in Ethiopia, in occupational therapy or clinical research nursing in Kenya) and received CST training directly from the WHO CST team. Just after the trial’s start, the competence of non-specialist facilitators leading home visits will be assessed using the WHO CST Adult–child Interaction Fidelity rating scale. Throughout the trial, the supervisors will observe three out of the nine group sessions, during which they will rate the facilitators’ competency and fidelity using the ENACT and ACC and record other session-related observations. CST supervisors will provide both individual and group feedback to the non-specialist facilitators about the CST intervention’s delivery. Facilitator and observer feedback forms, ACC, and ENACT data will be used as resources for supervision, including exploration of non-specialist facilitators’ experiences. Supervision record forms will be used to document the supervision. Monthly in-country and cross-country supervision coordination meetings will be held to ensure the CST intervention is implemented consistently across sites and to allow for discussion of common challenges observed across sites.

In Ethiopia, during home visits caregivers will be requested permission to record the facilitator interacting with the child. Through viewing these recordings and providing feedback based on these recordings, supervisors can support non-specialist facilitators in ensuring the CST goals set for the individual family align with the child’s developmental level and that the facilitator implements the CST strategies well with individual children. Security concerns preclude taking video recordings in Kenya. In the Kenyan sites, supervisors will join the facilitator to attend a subset of the home visits. Lastly, we define adherence to the CST intervention as the family participating in at least 7 group sessions and 2 home visits. We will monitor and promote adherence by organising CST group sessions in central, accessible locations, taking attendance at each group session, providing childcare support when caregivers attend sessions with their children, following up immediately with caregivers if group sessions or home visits are missed, and where necessary, by sending reminders before each scheduled activity.

### Relevant concomitant care permitted or prohibited during the trial {11d}

Families that previously participated in the CST intervention in Ethiopia and Kenya will be excluded from the trial (see exclusion criteria outlined above). Throughout the trial, caregivers will be encouraged to seek medical support or other locally available support (including any locally available education support), as needed. Our team has prepared site-specific resource kits for families to encourage help-seeking.

### Provisions for post-trial care {30}

Supports and resources specific to the trial, such as free child diagnostic assessments by clinical specialists and reimbursements for travel costs to health centres for project-related assessments, will be suspended after the trial ends. However, trained community support workers and health and education workers will retain the ability to identify, refer, and assess children suspected of having developmental disabilities. Caregivers will also receive site-specific resource lists with information about relevant local resources and services that will continue to provide care after the trial’s termination.

### Outcomes {12}

There are two co-primary outcomes: (1) the frequency of a child’s internalising and externalising problem behaviours measured using total raw scores of the preschool and school-age versions of the Child Behavior Checklist (CBCL) [24–27] and (2) the quality of life of the child’s family measured using total scores of the family impact module of the Pediatric Quality of Life (PEDsQL) measure—acute version, asking about the family’s quality of life across the past 7 days [28]. Both measures are caregiver-reported and will be assessed at T0 (baseline), endpoint (T1, 18 ± 2 weeks after randomisation), and follow-up (T2, 44 ± 2 weeks after randomisation). The primary time point of interest for the primary outcome variables is T1.

There are 12 secondary outcomes.

We have prioritised the PEDsQL and CBCL assessed at T2 as our most important secondary outcomes, to examine if any effects are sustained at follow-up. There are ten additional secondary outcomes which include:

1. Caregivers’ experience of stigma measured with an adapted version of the family interview schedule [29], given the high rates of stigma and discrimination experienced by families of children with DDs in these contexts [10, 30].
2. Caregivers’ internalisation of stigma related to their child’s condition using an adapted version of the affiliate stigma scale [31], given the tendency for caregivers to self-stigmatise based on their experiences with their child [32].
3. Caregivers’ depressive symptoms using the Ethiopian validated version of the Patient Health Questionnaire (PHQ-9) [33, 34], given extant evidence on the link between stress among caregivers of children with DDs and depressive symptoms [35, 36].
4. Caregivers’ knowledge and competencies relating to CST strategies using the WHO CST knowledge and skills checklist based on the perceived critical role of CST strategies in reducing child externalising and internalising problems [22].
5. Caregivers’ quality of life with the EuroQol 5 Dimensions 5 Levels (EQ-5D-5L) [37].
6. Children’s quality of life using a child proxy version of the EQ-5D-5L measure, referred to as the EuroQol 5 Dimensions Youth (EQ-5D-Y) [38]. To be used only in Kenya, because no validated version approved by the licence holder is available for use in Ethiopia.
7. Caregiver’s perspective of the family’s access to food using the Household Food Insecurity Access Scale (HFIAS) [39], considering the evidence demonstrating that caregiving responsibilities often prevent caregivers from engaging in viable economic activities and lead to economic hardship [35, 40].
8. Caregivers’ reported use of physical discipline on their child using the discipline section of UNICEF’s Multiple Indicator Cluster Survey (MICS) [41] based on reports from our pilot studies indicating reductions in caregivers’ use of physical punishment on their children with DDs [10].
9. Caregivers’ reported level of social support using the Oslo Social Support Scale [42] based on reports of social isolation experienced by caregivers of children with DDs and the CST’s group discussion format that brings caregivers together and promotes engagement among families with similar characteristics and experiences [43].
10. Caregivers’ report of their child’s mode and function of communication using an adapted version of the communication profile [44] based on the CST’s purported goal to increase communication and engagement in children with DDs [9].

All outcomes will be assessed at T0 (baseline), T1 (18 ± 2 weeks—in other words, around 18 × 7 = 126 days or ~ 4 months after randomisation), and T2 (44 ± 2 weeks—in other words, around 44 × 7 = 308 days or ~ 10 months after randomisation). Two of these additional outcomes, the EQ-5D-5L and the EQ-5D-Y, are only collected in the context of the health resource impact and cost and consequence analysis and will not be reported in the main trial paper. All other outcomes will be reported in the main trial paper. The main evaluations focus on the primary outcome measures at T1, the primary outcome measures at T2, and the secondary outcome measures at T1. Analyses of the remaining eight secondary outcome measures at T2 will be exploratory only and may or may not be reported in the main trial paper. The pre-specified exploratory moderation analyses will be reported in the main trial paper. The health resource impact and cost and consequence analysis and the mediation analyses will not be reported in the main trial paper.

### Participant timeline {13}

See Appendix Table 2.

### Sample size {14}

Each study cluster comprises 7 to 10 families. In intervention clusters, these families will all participate in the same CST group (with control clusters offered the intervention after data collection has been completed). Following WHO’s recommendations, CST groups will have a maximum of 10 caregivers. We anticipate having higher recruitment rates in the urban areas (i.e. 10 caregivers per cluster) compared to the rural areas (i.e. 7 caregivers per cluster), with an overall anticipated average cluster size of 8.5 caregivers.

Using a simulation and non-central chi-square approach [45], we determined that 14 clusters at each site (*n* = 476 caregivers in total) provided 96% (if effect size = 0.4) and 83% (if effect size = 0.32) power to detect the intervention effect for each co-primary at T1. The method accounted for (1) the cluster design, (2) the two co-primaries using a Bonferroni correction (i.e. alpha = 0.025), (3) realistic patterns of drop-out over repeated measures (i.e. 10% each at post-intervention and follow-up), (4) a missingness at random assumption, (5) pilot-test informed (i.e. 0.4) and conservative effect sizes (i.e. 0.32), and (6) intra-class correlation of 0.05 in our intervention arms. The random effects analysis model also co-varied for baseline and the country/site strata, and allowed for different group random effect variances for intervention and control arms, and different effects at T1 and T2 (i.e. including an intervention arm by time point interaction term in the model, with the effect at T1 extracted as the primary outcome). Furthermore, as this is a psychosocial trial with potential contamination concerns, we increased the number of trial clusters to 16 instead of 14 per site, bringing the total sample size to approximately 544 (i.e. an average of 8.5 families per cluster in 64 clusters) (Table 1).

**Table 1:** Ethics approval information from collaborating institutions

Country	Name of ethics committee and/or governing body	Reference number	Status
UK	Health Faculties (purple) Research Ethics Subcommittee-King’s College London	RESCM-23/24–33742	Approved
Ethiopia	Scientific and Ethics Review Committee-Centre for Innovative Development and Therapeutic Trials for Africa (CDT Africa)	CDT/5564/22	Approved
	Institutional Review Board (IRB)-Addis Ababa University College of Health Sciences	AAYMF 03–008	Approved
Kenya	Institutional Scientific and Ethics Review Committee (ISERC)-Aga Khan University, Nairobi	2021/IERC-99 (V4, 26-Feb-2024)	Approved
	Kenya Medical Research Institute Scientific and Ethics Review Unit (SERU)	KEMRI/SERU/CGMR-C/272/4872 (V1.3, 26-Jan-24)	Approved
	National Commission for Science, Technology and Innovation (NACOSTI), Kenya	NACOSTI/P/24/33550	Approved

### Recruitment {15}

The primary recruitment strategy will be to use a community-based identification tool, developed by our research team and implemented by trained community support workers in the local communities, to select children identified as potentially having developmental disabilities. Awareness-raising training with community stakeholders (e.g. local leaders, religious leaders) will also be conducted in an effort to reduce stigma and create an enabling environment for the identification and engagement of children with DDs and their families. Identified children will be referred to health or education workers trained using principles from WHO’s mhGAP [22] for further assessment. Two- to nine-year-old children with a positive assessment (i.e. probable DD based on mhGAP assessment) will be referred to a specialist clinician (e.g. psychiatrist or clinical officers with specialist training) for a more comprehensive assessment to confirm or negate the diagnosis. Subsequently, the caregivers of children with a confirmed DD diagnosis will be contacted by our research assistants to assess their eligibility based on all inclusion and exclusion criteria and obtain consent if they are eligible and willing to participate in the trial.

If needed, to achieve our desired sample size, we will also recruit eligible participants from educational institutions, health centres, specialist clinics, and Kenyan Education Assessment and Resource Centres. Administrators of these centres, who are gatekeepers to the participants, will share information about the study with families living in the SPARK clusters with a 2–9-year old child suspected of having a DD to assess preliminary interest. Interested potential participants will be contacted by members of our research team to provide a referral for a formal diagnostic assessment if they are recruited from non-specialist health centres or educational institutions and lack a formal diagnosis. Upon receiving a formal diagnosis, they will be contacted again by our research team to assess their eligibility based on all inclusion and exclusion criteria and obtain consent if willing and eligible. Non-eligible families from either route will be referred to local health centres and provided with a resource kit containing information about relevant local resources and services in their areas.

## Assignment of interventions: allocation

### Sequence generation {16a}

We will use the bespoke randomisation system maintained by the King’s Clinical Trial Unit (KCTU) at King’s College London’s to design a web-based randomisation system for participant allocation. Clusters will be randomised in randomly permuted blocks of size two, stratified by site (Addis Ababa, Gurage/Silte, Nairobi, and Kilifi). The system will require a minimum of two clusters to be randomised within a given site at a time, to maintain allocation concealment. In extreme circumstances where, after all conceivable efforts to recruit have been exhausted, we only have a single cluster to randomise within a site, we will generate a dummy cluster that will be entered into the randomisation system to permit random allocation of the single cluster.

### Concealment mechanism {16b}

Country-specific authorised staff (data managers independent from the SPARK research or clinical teams) will conduct randomisation using KCTU’s online randomisation system. The co-principal investigator (co-PI) or delegate (e.g. trial manager) will request non-shareable, user-specific usernames and passwords from the KCTU for authorised staff to access the system. Authorised staff will randomise clusters by going to www.ctu.co.uk and clicking on the link to access the randomisation system. Site-specific cluster numbers will be entered into the randomisation system. The system maintains a full audit trail of all data entered, including the staff who entered the data and a timestamp of the entry. As per ‘Sequence generation {16a}’ section, clusters will be randomised in pairs to conceal allocations.

### Implementation {16c}

Research staff will enrol participants in each cluster. This will involve screening caregivers and their eligible child and consenting eligible and willing caregivers. Subsequently, baseline assessments will be conducted with consenting families in each cluster once the target number of caregivers is attained (i.e. 7–10). Then, a country-specific independent allocator will use the randomisation system to assign clusters to the intervention or control arm and inform a SPARK research staff member, who will inform participants of their allocation status.

## Assignment of interventions: masking

### Who will be masked {17a}

Independent allocators will assign clusters to the intervention or control arm using the bespoke randomisation system designed by the King’s College Trial Unit. As such, they will be unmasked to cluster allocations but will have no interactions with participants. The site-specific data managers will be unmasked to facilitate routine database checks but will have no access to research participants and will not summarise or analyse data. Data collectors will be masked to allocation: research participants will be asked to conceal their allocation from the data collector. Data collectors will be systematically asked following T1 and T2 assessments about any accidental unmasking. The primary outcome measures will be collected first to reduce the impact should data collectors be unmasked during data collection. Measures to be completed exclusively by participants in the intervention arm will be completed during the CST sessions to ensure outcome data collectors remain masked during trial assessment time points.

The trial statistician will be masked to participant allocation until the statistical analysis plan (SAP) has been finalised and signed. The senior statistician will remain masked throughout the trial but will be unmasked to complete final checks on the analysis code and statistical report after data have been collected, the database has been locked, analysed, and the first draft of the statistical report has been prepared. The principal investigators, co-investigators, and trial steering committee members will also remain masked throughout the trial, except in a serious adverse event (SAE) that necessitates revealing a participant or specific cluster’s allocation. The level of masking of the DSMB will be at their discretion, but they will likely see data split across multiple clusters at least at a partially masked (i.e. arms described as A/B) level. The Trial Steering Committee (TSC) will only be provided with an open report with pooled data that is not separated out by arm. In case of a major safety concern, the DSMB and TSC can request unmasking. Masking will be maintained across these team members by preventing interactions with research participants. Further, masked team members will not have access to the extracted randomisation list or data summarised across clusters.

### Procedure for unmasking if needed {17b}

If needed, we will first unmask the trial statistician. The unmasked statistician will inform the DSMB of sections of the report pertaining to the different arms. In case of a major safety concern, the DSMB and TSC can request unmasking.

## Data collection and management

### Plans for assessment and collection of outcomes {18a}

Primary and secondary outcome data will be collected by trained data collectors. Other data (e.g. process data, clinical data) will be collected by data collectors, research assistants, and, in some cases, the supervisors and non-specialist facilitators during the CST sessions in each site. Details on data accuracy checks and procedures are outlined in the section below and in the data management plan. The different types of measures and forms that will be used in this study are listed and described below.

#### Demographic and service history information survey

This survey was developed by our research team to collect demographic and service history information like the receipt of educational and health services related to the child, the caregiver, and their household.

#### Developmental and clinical assessment

We will record the outcome of the developmental and clinical assessment, noting the type and severity of DD and any co-occurring conditions.

#### Two primary outcome measures

The PEDsQL. The PEDsQL Family Impact module is a 36-item scale measuring the impact of a child’s condition on the family’s functioning across eight domains: caregiver’s physical, emotional, social, and cognitive functioning, communication and worry, and the family’s daily activities and family relationships. The PEDSQL acute version, which asks about the family’s quality of life over the past 7 days, will be used. This instrument was previously adapted and translated into Amharic and has good to excellent ratings on internal consistency and test–retest reliability (i.e. 0.80–0.96) on the total scores and almost all subscales, and good known group validity [24, 28].

The CBCL. The 99-item preschool and 113-item school-age versions of the CBCL will be used to assess the child’s internalising and externalising behaviours. Caregivers rate the veracity of statements on three levels: 0—not true, 1—somewhat or sometimes true, and 2—very true or often true. The CBCL has been translated into many languages and validated worldwide, including Amharic and Swahili. In previous studies, the translated Swahili version was found to have excellent Cronbach alpha internal consistency ratings of 0.96 [25, 46, 47].

#### Additional secondary outcome measures

In addition to assessing the co-primary outcomes at T2, further secondary outcome measures include the following:

- The adapted affiliate stigma scale. The 22-item questionnaire assesses caregivers’ self-stigmatisation based on their child’s disability, on a four-point scale (i.e. 1—strongly disagree, 2—disagree, 3—agree, and 4—strongly agree) [31]. It had excellent internal consistency rates (i.e. Cronbach’s *α* = 0.95 and 0.94) when used among caregivers of people with intellectual disability and mental illness, respectively [31].
- The adapted family interview schedule. A 14-item measure assessing caregiver’s community-based experience of stigma, previously adapted and tested in caregivers of children with DD by our research group in Ethiopia [30]. Items are rated on four levels: 0—not at all, 1—sometimes, 2—often, and 3—a lot. The adaptation targeting caregivers of children with developmental disabilities was found to have a Cronbach alpha internal consistency rating of 0.92 [30].
- The Patient Health Questionnaire, nine-item version (PHQ-9). The PHQ-9 comprises nine questions assessing caregiver’s depressive symptoms and has been translated and validated for use in Ethiopia and Kenya [33, 48, 49]. We will use the Ethiopian adaptation, which first probes the presence of each symptom using a dichotomous variable (i.e. yes or no) and subsequently asks about the weekly frequency if a ‘yes’ response was endorsed. The PHQ-9 has shown evidence of good construct and convergent validity in previous studies [33].
- The Household Food Insecurity Access Scale (HFIAS). The HFIAS is a 9-item caregiver-reported measure assessing problems regarding access to food within the caregiver’s household over a 4-week period [39, 50]. The HFIAS has been translated and validated in Amharic and Swahili. Studies assessing the validity of the Ethiopian adapted version indicate the tool has adequate validity (i.e. *α* ranging from 0.73 to 0.76 in different rounds of tests).
- UNICEF’s Multiple Indicator Cluster Survey (MICS) on child discipline [41]. The measure explores parents’ perceptions of the value of punishment in raising children and the types of punishment they adopt. It includes 10 questions assessed on a dichotomous scale (i.e. yes or no).
- The WHO CST knowledge and skills checklist. This was developed specifically for the CST intervention and assesses caregivers’ knowledge of the CST (24 items) and their confidence in applying its strategies in routine care (13 items). Although it has been used extensively in CST studies [15, 21, 22], it has not yet been formally validated. Our team has translated the tool into Amharic and Swahili and will validate the measure in the trial baseline data.
- The EQ-5D-5L. This is a standardised measure for assessing caregiver’s quality of life in terms of the degree of problems faced along five dimensions: mobility, self-care, usual activities, pain/discomfort, and anxiety/depression. The degree of problems may be rated as no, slight, moderate, severe, or extreme. The tool has been widely used internationally and has shown moderate to strong correlations with global health measures [51]. We will use the approved Amharic and Swahili versions.
- The EQ-5D-Y. This is a proxy measure of the EQ-5D-5L for assessing children and adolescents’ quality of life in similar domains as the EQ-5D-5L. Items are rated on three levels—no problems, some problems, and a lot of problems. An approved Amharic version of the tool does not yet exist. Therefore, we will only use the currently available Swahili version in Kenya.
- The Oslo social support survey (OSSS-3). The OSSS-3 comprises three items assessing caregiver-reported social support along three dimensions: the caregiver’s number of close confidants, their perceptions of people’s concerns, and the ease of obtaining practical assistance. The tool has been used in previous studies with Ethiopian communities [52].
- The adapted communication profile. Children’s communication mode and function will be assessed using two validated subscales of the adapted communication profile, originally developed for use in Kenya and Uganda [53] and subsequently adapted, translated, and validated for use in Ethiopia [44]. The 33 items comprising the validated subscales are measured on five levels ranging from 0—never to 4—always. These subscales have excellent validity and reliability in Ethiopia (Cronbach’s alpha = 0.97; kappa = 0.60–0.95, *p* < 0.001; ICC = 0.97, *p* < 0.001) [44].

#### Other measures—costing tools

Client service receipt inventory (CSRI). We adapted this measure to assess caregivers’ reported costs associated with caring for a child with DD in the Kenyan and Ethiopian context and to capture costs associated with care and education for children with DD and caregivers’ health-service-related costs. The costing tool has been pilot-tested in both countries and will be collected at baseline (T0), endpoint (T1), and follow-up (T2). In addition, we will assess the CST intervention-related costs in the intervention arm only through a brief survey administered during one CST group session and one home visit.

Provider-related costs. We developed an activity-based costing tool to assess provider-related costs associated with administering the intervention. These include staff, supervision, training, equipment, and travel costs. This tool tracks costs throughout the trial and includes start-up costs.

#### Other measures—CST materials

Developmental assessment-goal-setting. The goal-setting form was developed by WHO to assess the child’s developmental level, specifically communication and play, and inform the setting of developmentally appropriate targets.

Home visit attendance and records of the facilitator-child interaction. Caregivers’ attendance at home visits will be recorded by the research team to track adherence. In Ethiopia, we will also video-record the interactions between non-specialist facilitators and children during selected home visits. The recordings will be used in supervisory meetings to further coach and guide the efforts of non-specialist facilitators. Security and logistical challenges prevent recording of sessions in Kenya, where instead, supervisors will join the facilitator to attend a subset of the home visits.

Experience with caregiver interventions survey. We developed this survey to ascertain caregivers’ exposure to interventions for children with DDs and caregiver training materials relevant to DDs via parent support groups, friends or family, services (education, health, NGOs), online, radio or TV, or booklets. This survey is administered at the follow-up assessment to assess possible contamination affecting the trial.

The WHO CST Adult–Child Fidelity (FCI) rating scale. This scale, developed by the WHO CST team, will be used to assess the competency of non-specialist facilitators in applying the CST strategies during their interactions with children just after the trial’s start. It includes 12 items assessing mastery of the CST intervention strategies to promote communication, regulated behaviour, and skills learning. Items are rated on a 5-point scale ranging from 0 (‘strategy not demonstrated or inappropriately applied—needs substantial improvement’) to 4 (‘strategy applied appropriately at least 80% of the time—very good implementation’).

The adapted ENACT [23]. This measure will be used to assess the competency of non-specialist facilitators in delivering CST group sessions during the trial. This is an 18-item questionnaire [22] rated on a 4-point scale ranging from 1—needs improvement to 4—done well. The ENACT will be rated by trained CST supervisors during 3 out of 9 CST group sessions.

The Activity Completion Checklist (ACC). This tool measures the fidelity of non-specialists to the CST group sessions manual and was developed by our team during the Ethiopian pilot test. It indicates whether each activity in the session manual was fully, mostly, partly, or not completed. This checklist will also be rated by trained CST supervisors during 3 out of 9 CST group sessions.

CST group session attendance and home practice completion. We will maintain a record of caregivers’ attendance at the CST group sessions to ascertain adherence. Although we actively encourage only the consenting caregiver to participate in the CST sessions, we recognise that caregiving is socially distributed [54] and will record instances where there is a change in the caregiver attending specific CST sessions. Home practice completion will also be recorded during each group session.

In-depth interviews and focus group discussions. After all follow-up data collection is completed, focus group and in-depth interviews will be held with CST non-specialist facilitators, CST supervisors and master trainers, and caregivers. These topic guides explore how well the CST worked and consider contextual barriers and facilitators that may have influenced implementation, with a special focus on long-term sustainability. The full range of contextual determinants (informed by implementation science determinant frameworks), implementation outcomes, and process measures will be described in a separate implementation science protocol paper.

### Plans to promote participant retention and complete follow-up {18b}

We will promote participant retention by organising CST group sessions in central locations accessible to all group members. Guided by local research guidelines and practice, in Kenya participants will be compensated for research activity including travel time, will be given a refreshment/meal, and be compensated for out-of-pocket expenses and travel costs. In Ethiopia, participants will be given refreshments during all CST group sessions and will be reimbursed for travel costs.

If participants need to travel for collection of research data, they will be reimbursed for travel costs and compensated for time. If research data collection is at the participant’s home, they will receive an in-kind recognition of time burden (0.5 kg coffee in Ethiopia, dry food stuffs in Kenya).

Regardless of intervention adherence, the primary caregiver who completed the T0 assessment will be invited to participate in the T1 and T2 assessments. Likewise, caregivers in the control arm who are exposed to the CST intervention (through study error, trial contamination or due to exposure to the CST outside the trial) will remain in the trial control arm and will have their outcomes assessed.

### Data management {19}

The latest version of the Research Electronic Data Capture (REDCap) [55, 56] clinical data management system hosted at Addis Ababa University will be used in this trial. Data for the primary and secondary outcomes will be entered directly into REDCap as source data, with the exception of the EQ-5D measures, which stipulate paper and pen data collection and will be entered into REDCap immediately after data collection. Some process measures and key clinical information from the clinical verification assessment are collected on paper and subsequently entered in the electronic Case Report Form. Each site will have rights to access, view, download, and conduct routine internal checks on their site-specific data. Data entered from paper records entered in REDCap will be checked against the source data for accuracy. All data will be centrally stored and managed by the AAU trial team at the Clinical Trial Unit at the Centre for Innovative Drug Development and Therapeutic Trials for Africa (CDT-Africa). The AAU trial team will complete further checks based on the detailed data management plan. Data will be locked for analysis after all data have been entered, checks have been completed, and queries from audit checks, routine monitoring, and external audit checks have been addressed.

Paper source copies of process data will be stored for a minimum of 10 years in secure filing cabinets in each site’s project coordinator’s office. Other electronic data such as qualitative process data will be stored on encrypted project laptops and backed up on encrypted project hard drives and/or automatically on university servers at local sites. Pseudonymised qualitative data will be stored centrally in the project’s SharePoint data storage system: a GDPR-compliant file-sharing system managed by King’s College London and accessible to all collaborators.

### Confidentiality {27}

All research staff will be trained on the importance of ensuring participant and data confidentiality.

Participant consent will be carried out in private areas where they can comfortably consider participating without intrusion. However, the potential participant can consult with others before deciding to participate. Once enrolled, they can also object to participating in specific activities (e.g. video recordings) or withdraw from the study at any time.

All primary data collected in this project will be stored securely in the collecting institution following institution-specific data governance policies. All participating families will be assigned a caregiver-child dyad identification (ID) number. Data files will include only the ID code without any personal identifiable information. We will maintain a single master file per site linking caregiver-child dyad IDs with personal identifiers in a secure location in the in-country PI’s, trial coordinator’s, or data manager’s office. This file will not be stored centrally and will be maintained and updated by the in-country PI, site-specific trial coordinator, or data manager. The primary storage location for paper-based data will be a secure filing cabinet in the local site’s research office, and paper-based data with personal identifiers (e.g. consent forms) will be kept separate from questionnaire and interview data.

Electronic data will be collected using project-encrypted laptops and stored on the local site-specific servers and, subsequently, on the central server or network. In Ethiopia, external hard drives will also be used to back up electronic data regularly.

### Plans for collection, laboratory evaluation, and storage of biological specimens for genetic or molecular analysis in this trial/future use {33}

Not applicable, as we will not be collecting biological specimens.

## Statistical methods

### Statistical methods for primary and secondary outcomes {20a}

Primary and secondary outcome variables will be summarised using appropriate descriptive statistics (mean and standard deviation, median and interquartile range or frequency and proportion overall and by intervention arm at baseline (T0), and at endpoint (T1) (18 ± 2 weeks post-randomisation) and follow-up (T2) (44 ± 2 weeks post-randomisation)).

The primary analysis will be an individual-level analysis conducted using separate multilevel, mixed-effects linear regression models for each of the primary outcomes, with the two post-randomisation measures as dependent variables, and adjusting for baseline score, time point and country/site strata as fixed effects. Models will include an intervention arm-by-time point interaction to allow for different effects at T1 and T2, with the effect at T1 extracted as the primary outcome. It will also include pre-specified adjustments for the baseline variables child age, child sex, and caregiver education to reduce the impact of any imbalance of potential confounders at the individual level between arms. We will plot normality probability graphs of the residuals from the primary analysis models to assess the degree to which scales conform to model assumptions, with either normalising transformations applied or robust standard errors used where necessary. Tests of intervention effect homogeneity by country will be presented. If the country effect is significant, we will subsequently assess intervention effect homogeneity by site (urban vs rural) within country. The administration of two different CBCL questionnaires to children of different ages will be handled by estimating Cohen’s *d* effects for the questionnaires separately, using the baseline standard deviation in each case, and then combining the two Cohen’s *d* estimates together as a weighted average with an appropriate confidence interval.

We will use similar methods to analyse the secondary outcomes. The main secondary outcomes will be the intervention effect on PEDsQL and CBCL at T2, extracted from the models described for the primary outcomes. All other secondary outcomes are also continuous variables and will be analysed using similar models, using the T1 intervention effect extracted for each secondary outcome. A detailed statistical analysis plan will be drafted and approved by the DSMB.

### Interim analyses {21b}

No interim analyses have been planned.

### Methods for additional analyses (e.g. subgroup analyses) {20b}

We will carry out exploratory pre-specified analyses to determine whether specific baseline moderators, i.e. (a) caregiver educational level; and (b) social support, modify the intervention’s effect on children’s behavioural problems (CBCL) and caregivers’ quality of life (PEDsQL) at T1 and T2.

We will also assess mediational and post-randomisation moderator effects, which will not be reported in the main trial paper. The mediational analysis will use the interventional in(direct) effects approach [57] that permits simultaneously accounting for post-randomisation moderators and multiple mediators. With it, we can investigate the extent to which the intervention induced change in caregiver quality of life by changing child behavioural problems (our main mediator). The method will also reveal how several indirect effects contributed to the intervention’s total effect. We plan to investigate the following: (i) the indirect effect of the intervention on change in child behavioural problems; (ii) the indirect effects through post-randomisation moderator factors such as the number of CST sessions attended, fidelity of CST group sessions and competence of the CST facilitator, and home practice completed; and (iii) the indirect effects of the intervention through other potential mediators such as social support, caregiver perceived stigma, and physical punishment.

### Methods in analysis to handle protocol non-adherence and any statistical methods to handle missing data {20c}

Our power and sample size calculations account for missing data based on realistic patterns of dropout over repeated measurements. We will produce frequency reports of participants who purposefully withdraw from the trial or are passively lost to follow-up by site and arm at 18 ± 2 weeks post-randomisation (T1) and 44 ± 2 weeks post-randomisation (T2).

We will use intention to treat principles that analyse participants within their randomised clusters, with mixed effects models including participants with at least one outcome measure, assuming data are missing-at-random. Our analyses will also include any baseline variables found to be associated with missing outcome data in models to make the missing-at-random assumption more plausible.

Moreover, if attrition rates exceed the levels assumed in the power calculation, we will compute a sensitivity analysis that will impute missing-data values under two scenarios: (i) outcomes remain unchanged from or return to baseline and (ii) where attrition in the control arm is related to improvement of 0.1 SD and in the active arm is related to a worsening of 0.1 SD [58, 59].

We define adherence as the family participating in at least 7 group sessions and 2 home visits, and if we register non-adherence rates of 10% or more, we will do a complier average causal effect (CACE) analysis for the two primary outcomes [59, 60] as a more principled version of a per-protocol analysis. We will compare those who complied/were adherent in the intervention arm with those in the control arm who would have been adherent if they had been allocated to the intervention arm. The CACE will be obtained using instrumental variable methods [60] with the primary outcome of interest as the dependent variable and adjusting for similar variables to those described for the primary intention to treat analysis. If we later find any individuals were ineligible and mistakenly entered into the trial, we may redo the CACE analysis with these individuals removed.

### Plans to give access to the full protocol, participant-level data, and statistical code {31c}

The full trial protocol and consent forms will be published on the Open Science Framework (OSF) website (https://osf.io/) after the trial protocol paper has been published. Data underlying the results reported in the main trial paper will be made available upon request 12 months after the main trial article has been published. Access to the data will be controlled by the SPARK executive group. Data sharing will be prioritised for teams involving students and researchers in Ethiopia and Kenya in line with the capacity-building aims of the SPARK research programme. We have not yet planned to grant public access to the statistical code.

## Oversight and monitoring

### Composition of the coordinating centre and trial steering committee {5d}

The trial will be supervised and managed by a Trial Management Group (TMG) assisted by country-specific Trial Management Teams (TMTs), a Data Safety and Monitoring Board (DSMB), and a Trial Steering Committee (TSC). The country-specific TMTs will comprise trial staff and will be responsible for country-specific ethics applications, recruiting, training and supervising data collectors, and monitoring the quality of incoming data under the authority and guidance of local coordinating centres and institutions. Their work will be monitored by the cross-country TMG to ensure consistency in procedures. The TMG will include psychiatrists, psychologists, statisticians, epidemiologists, and trial coordinators from Ethiopia, Kenya, and the UK. They will oversee data collection, documentation, management, and the creation of an electronic data capture using REDCap to ensure that high-quality relevant data is collected and retained to protect the participants’ rights.

The DSMB will comprise three members independent of the SPARK study, including clinicians and biostatisticians. The board will meet approximately every 6 months to review data reports from the trial detailing recruitment tallies, cluster randomisation, CST participation and home visit completion rates by cluster and site, progress with data collection and entry at baseline, endpoint, and follow-up time points, and the number and types of SAEs and adverse events (AEs) reported per arm. Based on their review, they will recommend different actions, including pausing or ceasing the trial if irregularities are noted. Masking will be at the board’s discretion, and meetings may be closed or open to observers such as trial representatives.

The TSC will act on the sponsor and funder’s behalf to oversee the project. The committee will include six independent members (i.e. a chair, a statistician, two clinicians, and an informed community stakeholder) and non-independent members like the two project co-PIs, the Ethiopian in-country PI, and the other statisticians. The committee will meet approximately every 6 months, about 2 weeks after the DSMB’s meeting, to review, consider, and decide on recommendations in the DSMB report. Only independent members will have voting rights. Membership in the DSMB and TSC will be mutually exclusive and correspondence between the committees and the SPARK co-PIs will occur through their respective chairs.

### Composition of the data monitoring committee, its role and reporting structure {21a}

See ‘Composition of the coordinating centre and trial steering committee {5d}’ section above.

### Adverse event reporting and harms {22}

We have made provisions for detecting, recording, and managing AEs and SAEs. Adverse events include (i) potential adverse care of the child, including neglect, abuse, and chaining/restraint, (ii) deterioration in physical health or under nutrition of the child, (iii) caregiver suicidal ideation, (iv) stigma and discrimination, and (v) significant change or breakdown in family structure. Serious adverse events include (i) death due to suicide; (ii) death due to any other cause; (iii) suicide attempt; (iv) hospitalisation due to any medical or mental health emergency; (v) violence to others causing injury; and (vi) injury to the caregiver or child from others (life-threatening/requires hospitalisation/causes disability). Research staff may receive information about AEs and SAEs from caregivers, community members, health workers, and data collectors during systematic outcome assessment data collection efforts, trial-related appointment visits with health workers, or through caregiver-initiated reports to the research team outside scheduled visits. Research staff will report all suspected AEs and SAEs to the trial/project coordinator, who will inform the in-country PIs. The in-country PIs will assess the reports for relatedness, seriousness, and expectedness and determine whether the AEs need to be re-categorised as SAEs.

Within 24 h of the research team becoming aware of an SAE, the in-country PI will report the incident to all relevant Institutional Review Boards (IRBs), their internal monitors, and the co-PI at the sponsor institution. The co-PI at the sponsor institution will, in turn, report the incident to the DSMB, sponsor, and ethics committee at the sponsor institution within the same 24-h period of the research team becoming aware of the incident. The in-country-PI will complete and submit the more detailed SAE form within 8 days from the date of awareness to the co-PI at the sponsor institution for authorisation. Within 10 days from the date of awareness, the co-PI at the sponsor institution will submit the authorised SAE form using a secure medium to the DSMB, the sponsor and ethics committee at the sponsor institution, and in-country PI for onward submission to the relevant IRBs and internal monitors. Where the co-PI at the sponsor institution or the in-country PIs are unavailable, delegated staff members will take on these responsibilities.

All AEs and SAEs will be documented on AE/SAE forms and entered in the register of SAEs, which will be managed centrally by the trial coordinator. Tallies of all AEs and SAEs will be included in six-monthly reports to the Data Safety Monitoring Board (DSMB) and Trial Steering Committee (TSC). SAEs will be addressed on a case-by-case basis by members of the DSMB.

Guidelines on how to manage each SAE and AE are detailed in a dedicated standard operating procedure. In brief, all caregivers or children in the intervention and control arms experiencing SAEs/AEs will receive a phone call (for AEs only), visit, or invitation for assessment from CST non-specialist facilitators or research team members (i.e. trial or project coordinators or data collectors). They will be provided with further access to psychosocial or medical assistance based on their needs. Research team members will follow up with the caregiver as needed. Where the SAE/AE hinders a caregiver’s ability to participate in the trial, they will be invited to return, if desired, once the SAE/AE has been sufficiently managed.

### Frequency and plans for auditing trial conduct {23}

We will engage independent country-specific trial monitors with experience in trial monitoring and Good Clinical Practice to review the approved study protocol and conduct at least three site visits for audit purposes. The visits will occur before recruitment begins, at the mid-point, and at the end of the trial.

### Plans for communicating important protocol amendments to relevant parties (e.g. trial participants, ethical committees) {25}

The trial management group and site-specific trial management teams will communicate all important protocol modifications to relevant parties such as the interventionists, investigators, ethics committees/IRBs, trial participants, monitoring committee members, trial registries, and journals.

### Dissemination plans {31a}

We will prepare different kinds of outputs to communicate the trial’s results to researchers and stakeholders such as representative organisations for people with DD and their caregivers, policymakers, representatives from national and international non-governmental organisations, members of our national and community advisory boards, media representatives, and the public. Examples of the types of outputs we will prepare include peer-reviewed manuscripts, oral and poster presentations, flyers, toolkits, theory of change maps, policy briefs, and press releases.

All outputs will be made available on the SPARK website (https://www.thesparkproject.net/). In addition, we will organise specific events like social science cafes and project and community advisory board meetings to discuss these outputs and directly engage with stakeholders. We will use a targeted approach in developing and distributing the outputs to ensure stakeholders receive them in preferred formats and at an opportune time to increase the chances of uptake.

## Discussion

The WHO CST programme has previously been adapted and pilot-tested in field trials in 30 + countries [7]. The findings from these early studies suggest that it may be an acceptable, feasible, and effective intervention for children with DDs such as autism and intellectual disability and their caregivers. However, these investigations have been limited by several factors such as small sample sizes, delivery by specialists instead of non-specialist facilitators, a focus on autistic children only, and limited implementation in LMICs [17, 19, 21]. This study addresses these gaps through the conduct of a well-powered large trial and the generation of evidence of the effectiveness of the non-specialist-delivered version of CST in improving the mental health and well-being of children with DDs and their caregivers in two LMICs. The trial will also help to determine the resource impacts and costs and consequences associated with the intervention. This project aligns with the recently published WHO UNICEF Global report on children with developmental disabilities, which called for action to strengthen services, address stigmatisation, and inform, empower, and support caregivers of children with DD [1].

Despite the trial’s importance and potential positive impact, there are some ethical concerns to consider. First, participating in this trial may expose the child’s condition to community members, potentially increasing the risk of experiencing stigma and discrimination. We have minimised this risk by employing community sensitisation efforts in each of the trial areas, raising awareness of DDs, addressing negative beliefs about children with DDs in the community, and training staff in the importance of maintaining confidentiality. In addition, all trial workers received training in how to address stigma when it occurs and how to minimise the risk of stigma by ensuring participant confidentiality at all times. Secondly, participation in the CST programme may raise caregivers’ hopes for a cure for their child’s condition that cannot be met in this trial and are unrealistic given the chronic nature of their children’s conditions. The information sheet and consent process explicitly address this expectation of a cure, and this topic is also addressed during CST group sessions.

The WHO CST intervention targets children aged 2–9 years with developmental delays in the social-communicative domain. Our trial recruits participants through community identification. There will be children identified in the community needing support who do not meet the trial’s inclusion criteria (e.g. because they fall outside the target age) or meet one or more of the trial’s exclusion criteria (e.g. co-occurring severe motor impairments). Our trial is conducted in a context where support systems for these children may not be fully developed. To mitigate this ethical concern, we have ensured local health and/or education workers in each cluster are trained in principles of the WHO mhGAP developmental disorder module, which constitutes enhanced care as usual since this training is not typically available in Ethiopian and Kenyan communities. Moreover, we have developed site-specific resource kits describing relevant support services available in each community to assist families with relevant referrals.

This trial seeks to test the effectiveness of the WHO’s CST as delivered by non-specialist facilitators at improving the mental health and wellbeing of children with DD and their caregivers living in four sites across Kenya and Ethiopia, in contexts where formal support for these families is largely unavailable, and families are impacted by poverty, stigma, and social exclusion. The project will also test the resource impacts and costs and consequences of the intervention. The findings of this trial can inform future implementation and scale-up of intervention services for families with children with developmental disabilities in Kenya and Ethiopia, as well as inform implementation in other low-resource contexts.

## Trial status

Trial protocol version: version 5.

Date: 7th March 2024.

Anticipated trial recruitment date: 8th April 2024.

Anticipated completion date: 28th February 2026.

## Supplementary Information

Supplementary Material 1.

## Data Availability

Access to the data is controlled by the SPARK executive group. Investigators wishing to use the data reported in the main trial will complete a publication proposal form, with an abstract of the proposed work, data sources, timelines, authorship, and proposed dissemination. The proposal should be directed to Rosa.Hoekstra@kcl.ac.uk and Amina.Abubakar@aku.edu, who will circulate it to the SPARK executive group for approval. Proposals will be evaluated on the criteria stipulated in the SPARK publication plan hosted on the SPARK website: https://www.thesparkproject.net/.

## Appendix

**Table 2:** Participant timeline

	**Clinical assessment (by specialist clinician)**	**Study information and screening (by research staff)**	**T0, baseline assessment (pre-randomisation; by independent data collector)**	**Randomisation**	**CST home visit 1, 2, 3 (by CST non-specialist facilitators)**	**CST group session 1, 2, 3, 4, 5, 6, 7, 8, 9 (by CST non-specialist facilitators)**	**T1, endpoint assessment (by independent data collector)**	**T2, follow-up assessment (by independent data collector)**	**Qualitative assessment (by independent researcher)**
**Study week**	≈ − 5	≈ − 5 to − 2	− 2 to − 1	0	2; 8; 13	3–7; 9–12	18 ± 2 weeks	44 ± 2 weeks	Week 46–58
**Assessment/procedure**									
Provide Patient Information Sheet and Informed Consent Form		X							x
Obtain written informed consent		X							x
Basic demographic information relating to inclusion and exclusion criteria		X							
Demographic and service history information			x						
Medical history	x								
Physical examination	x								
Developmental and clinical assessment	x								
Primary outcome measures: - PEDsQL - CBCL			x				x	x	
Secondary outcome measures: - Adapted affiliate stigma scale - Adapted FIS stigma scale - PHQ-9 - HFIAS - Discipline section MICS6 - WHO caregiver knowledge skill test - EQ-5D-5L - EQ-5D-Y - Oslo Social Support Scale - 2 subscales communication profile-adapted			x				x	x	
Experiences with caregiver interventions survey								x	
Economic costing measures—general (assets, service use costs, etc.) unrelated to intervention			x				x	x	
Economic costing measures—caregiver costs relating to CST					x	x			
Developmental assessment and CST goal setting					x				
Home visit attendance					x				
Ethiopian sites only^*^: video recordings during home visit to aid CST supervision					x				
CST activity completion checklist (collected 3 out of 9 sessions)						x			
ENACT (CST competency delivering group sessions) (collected 3 out of 9 sessions)						x			
CST group session attendance						x			
Homework completion						x			
In depth interviews Focus group discussions									x

^*^Due to safety concerns, video recordings are not feasible in Kenya

## Abbreviations

AAU: Addis Ababa University
AE: Adverse events
CACE: Complier average causal effect
CBCL: Child Behavior Checklist
CDT-Africa: Centre for Innovative Drug Development and Therapeutic Trials for Africa
CST: Caregiver Skills Training
DD: Developmental disabilities
DSMB: Data Safety and Monitoring Board
ENACT: ENhancing Assessment of Common Therapeutic factors
EQ-5D-5L: EuroQol-5 Dimensions-5 Levels
EQ-5D-Y: EuroQol-5 Dimensions-Youth
HFIAS: Household Food Insecurity Assessment Scale
HIC: High-income country
ID: Identification
IRB: Institutional Review Board
KEMRI-WTRP: Kenya Medical Research Institute-Welcome Trust Research Programme
KCTU: King’s Clinical Trial Unit
LMIC: Low- and middle-income country
MISC: Multiple Indicator Cluster Survey
NSF: Non-specialist facilitators
NGO: Non-governmental organisation
OSF: Open Science Framework
OSSS: Oslo Social Support Scale
PEDsQL: Pediatric Quality of Life
PHQ-9: Patient Health Questionnaire-9
PI: Principal investigator
RCT: Randomised controlled trial
REDCap: Research Electronic Data Capture
SPARK: SuPporting African communities to increase the Resilience and mental health of Kids with developmental disorders and their caregivers
TMG: Trial Management Group
TMT: Trial Management Team
TSC: Trial Steering Committee
SAE: Serious adverse events
UNICEF: United Nations Children’s Fund
WHO: World Health Organization
