# Supplementary material for: Supporting African communities to increase resilience and mental health of kids with developmental disabilities and their caregivers using the World Health Organization’s Caregiver Skills Training Programme (SPARK trial): study protocol for a cluster randomised clinical controlled trial
Source: Trials. 2024 Oct 24;25:713. doi: 10.1186/s13063-024-08488-w (PMC11515546; doi:10.1186/s13063-024-08488-w)
Supplement: Supplementary file 1 — Supplementary Material 1. [file 13063_2024_8488_MOESM1_ESM.zip › CONSORT_2Oct24_in press.docx]

Table 1| CONSORT 2010 Checklist of Information to Include When Reporting a Cluster Randomised Trial

| **Section/topic and item No** | **Standard checklist item** | **Extension for cluster designs** | **Page No*** |
| --- | --- | --- | --- |
| Title and abstract |  |  |  |
| 1a | Identification as a randomised trial in the title | Identification as a cluster randomised trial in the title | 1 |
| 1b | Structured summary of trial design, methods, results, and conclusions (for specific guidance see CONSORT for abstracts) | See table 2 | 1&2 |
| Introduction |  |  |  |
| Background and objectives: |  |  |  |
| 2a | Scientific background and explanation of rationale | Rationale for using a cluster design | 4-7 |
| 2b | Specific objectives or hypotheses | Whether objectives pertain to the cluster level, the individual participant level, or both | 7 |
| Methods |  |  |  |
| Trial design: |  |  |  |
| 3a | Description of trial design (such as parallel, factorial) including allocation ratio | Definition of cluster and description of how the design features apply to the clusters | 7 |
| 3b | Important changes to methods after trial commencement (such as eligibility criteria), with reasons |  | No changes yet |
| Participants: |  |  |  |
| 4a | Eligibility criteria for participants | Eligibility criteria for clusters | Participants-7-9; Cluster 7 |
| 4b | Settings and locations where the data were collected |  | 7 |
| Interventions: |  |  |  |
| 5 | The interventions for each group with sufficient details to allow replication, including how and when they were actually administered | Whether interventions pertain to the cluster level, the individual participant level, or both | 9- 10 |
| Outcomes: |  |  |  |
| **Section/topic and item No** | **Standard checklist item** | **Extension for cluster designs** | **Page No*** |
| 6a | Completely defined prespecified primary and secondary outcome measures, including how and when they were assessed | Whether outcome measures pertain to the cluster level, the individual participant level, or both | 11- 13 |
| 6b | Any changes to trial outcomes after the trial commenced, with reasons |  | No changes yet |
| Sample size: |  |  |  |
| 7a | How sample size was determined | Method of calculation, number of clusters(s) (and whether equal or unequal cluster sizes are assumed), cluster size, a coefficient of intracluster correlation (ICC or k), and an indication of its uncertainty | 19 |
| 7b | When applicable, explanation of any interim analyses and stopping guidelines |  | None planned |
| Randomisation |  |  |  |
| Sequence generation: |  |  |  |
| 8a | Method used to generate the random allocation sequence |  | 20 |
| 8b | Type of randomisation; details of any restriction (such as blocking Details of stratification or matching if used and block size) |  | 20 |
| Allocation concealment mechanism: |  |  |  |
| 9 | Mechanism used to implement the random allocation sequence (such as sequentially numbered containers), describing any steps taken to conceal the sequence until interventions were assigned | Specification that allocation was based on clusters rather than individuals and whether allocation concealment (if any) was at the cluster level, the individual participant level, or both. | 20 |
| Implementation: |  |  |  |
| 10 | Who generated the random allocation sequence, who enrolled participants, and who assigned participants to interventions | Replaced by 10a, 10b, and 10c | 20 |
| **Section/topic and item No** | **Standard checklist item** | **Extension for cluster designs** | **Page No*** |
| 10a |  | Who generated the random allocation sequence, who enrolled clusters, and who assigned clusters to interventions | 20 |
| 10b |  | Mechanism by which individual participants were included in clusters for the purposes of the trial (such as complete enumeration, random sampling) | 20 |
| 10c |  | From whom consent was sought (representatives of the cluster, or individual cluster members, or both) and whether consent was sought before or after randomisation | 9 |
| Blinding: |  |  |  |
| 11a | If done, who was blinded after assignment to interventions (for example, participants, care providers, those assessing outcomes) and how |  | 21 |
| 11b | If relevant, description of the similarity of interventions |  | N/A |
| Statistical methods: |  |  |  |
| 12a | Statistical methods used to compare groups for primary and secondary outcomes | How clustering was taken into account | 26 |
| 12b | Methods for additional analyses, such as subgroup analyses and adjusted analysis. |  | 27 |
| Results |  |  |  |
| Participant flow (a diagram is strongly recommended): |  |  | N/A |
| 13a | For each group, the numbers of participants who were randomly assigned, received intended treatment, and were analysed for the primary outcome | For each group, the numbers of clusters that were randomly assigned, received intended treatment, and were analysed for the primary outcome | 43 |
| 13b | For each group, losses and exclusions after randomisation, together with reasons | For each group, losses and exclusions for both clusters and individual cluster members | N/A |
| Recruitment: |  |  |  |
| **Section/topic and item No** | **Standard checklist item** | **Extension for cluster designs** | **Page No*** |
| 14a | Dates defining the periods of recruitment and follow-up |  | 14-18 |
| 14b | Why the trial ended or was stopped |  | N/A |
| Baseline data: |  |  |  |
| 15 | A table showing baseline demographic and clinical characteristics for each group | Baseline characteristics for the individual and cluster levels as applicable for each group | N/A |
| Numbers analysed: |  |  |  |
| 16 | For each group, number of participants (denominator) included in each analysis and whether the analysis was by original assigned groups | For each group, number of clusters included in each analysis | N/A |
| Outcomes and estimation: |  |  |  |
| 17a | For each primary and secondary outcome, results for each group, and the estimated effect size and its precision (such as 95% confidence interval) | Results at the individual or cluster level as applicable and a coefficient of intracluster correlation (ICC or k) for each primary outcome | N/A |
| 17b | For binary outcomes, presentation of both absolute and relative effect sizes is recommended |  | N/A |
| Ancillary analyses: |  |  |  |
| 18 | Results of any other analyses performed, including subgroup analyses and adjusted analyses, distinguishing prespecified from exploratory |  | N/A |
| Harms: |  |  |  |
| 19 | All important harms or unintended effects in each group (for specific guidance see CONSORT for harms106) |  | 29-30 |
| Discussion |  |  |  |
| Limitations: |  |  |  |
| 20 | Trial limitations, addressing sources of potential bias, imprecision, and, if relevant, multiplicity of analyses |  | 31-32 |
| **Section/topic and item No** | **Standard checklist item** | **Extension for cluster designs** | **Page No*** |
| Generalisability: |  |  |  |
| 21 | Generalisability (external validity, applicability) of the trial findings | Generalisability to clusters and/or individual participants (as relevant) | N/A |
| 22 | Interpretation consistent with results, balancing benefits and harms, and considering other relevant evidence |  | N/A |
| Other information |  |  |  |
| Registration: |  |  |  |
| 23 | Registration number and name of trial registry |  | 2 |
| Protocol: |  |  |  |
| 24 | Where the full trial protocol can be accessed, if available |  | 28 |
| Funding: |  |  |  |
| 25 | Sources of funding and other support (such as supply of drugs), role of funders |  | 34 |
| *Page numbers optional depending on journal requirements. | | | |

Table 2| Extension of CONSORT for abstracts of cluster randomised trials

| **Item** | **Standard checklist item** | **Extension for cluster trials** |
| --- | --- | --- |
| Title | Identification of study as randomised | Identification of study as cluster-randomised |
| Trial design | Description of the trial design (for example, parallel, cluster, non-inferiority) |  |
| Methods: |  |  |
| Participants | Eligibility criteria for participants and the settings where the data were Eligibility criteria for clusters collected |  |
| Interventions | Interventions intended for each group |  |
| Objective | Specific objective or hypothesis | Whether objective or hypothesis pertains to the cluster level, the individual participant level, or both |
|  |  | Whether the primary outcome pertains to the cluster level, the individual participant level or both |
| Outcome | Clearly defined primary outcome for this report |  |
| Randomisation | How participants were allocated to interventions | How clusters were allocated to interventions |
| Blinding (masking) | Whether or not participants, care givers, and those assessing the outcomes were blinded to group assignment |  |
| Results: |  |  |
| Numbers randomised | Number of participants randomised to each group Number of clusters randomised to each group |  |
| Recruitment | Trial status* |  |
| Numbers analysed | Number of participants analysed in each group Number of clusters analysed in each group |  |
| Outcome | For the primary outcome, a result for each group and the estimated effect size and its precision | Results at the cluster or individual level as applicable for each, primary outcome |
| Harms | Important adverse events or side effects |  |
| Conclusions | General interpretation of the results |  |
| Trial registration | Registration number and name of trial register |  |
| Funding | Source of funding |  |
| *Relevant to conference abstracts. | | |
