# Supplementary material for: Supporting African communities to increase resilience and mental health of kids with developmental disabilities and their caregivers using the World Health Organization’s Caregiver Skills Training Programme (SPARK trial): study protocol for a cluster randomised clinical controlled trial
Source: Trials. 2024 Oct 24;25:713. doi: 10.1186/s13063-024-08488-w (PMC11515546; doi:10.1186/s13063-024-08488-w)
Supplement: Supplementary file 1 — Supplementary Material 1. [file 13063_2024_8488_MOESM1_ESM.zip › Sample consent form and information sheet_no contact details.docx]

**CONSENT FORM FOR PARTICIPANTS IN RESEARCH STUDIES**

1. **SAMPLE CONSENT FORM FOR CAREGIVERS’ PARTICIPATION IN RCT OF THE CST USED IN ETHIOPIA (NB Ethiopian participants receive the form below in Amharic translation; Kenyan participants receive a version in Kiswahili, with all forms referring to Kenyan institutions and institutional review boards)**

**Please complete this form after you have read the Information Sheet and/or listened to an explanation about the research.**


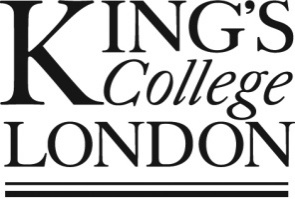

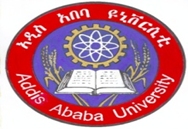


***Title of Study: SuPporting African communities to increase the Resilience and mental health of Kids with developmental disabilities and their caregivers (SPARK).***

**King’s College Research Ethics Committee Ref: ________________**

**Please tick or initial**

Thank you for considering taking part in this research. The person organising the research must explain the project to you before you agree to take part. If you have any questions arising from the Information Sheet or explanation already given to you, please ask the researcher before you decide whether to join in. You will be given a copy of this Consent Form to keep and refer to at any time.

**Please tick or initial**

**I confirm that I understand that by ticking/initialling each box I am consenting to this element of the study. I understand that it will be assumed that unticked/initialled boxes mean that I DO NOT consent to that part of the study. I understand that by not giving consent for any one element I may be deemed ineligible for the study.**

1. I confirm that the information sheet dated 23rd February 2024 version number 4 for the above study has been read to me/ by me and I understand its contents. I have had the opportunity to consider the information and asked questions which have been answered to my satisfaction.
2. I consent voluntarily to be a participant in this study and understand that I can refuse to answer questions and I can withdraw from the study at any time, without having to give a reason, up until ten months after this consent date.
3. I consent to the processing of my personal information for the purposes explained to me in the Information Sheet. I understand that such information will be handled in accordance with the terms of UK data protection law (including the UK General Data Protection Regulation (UK GDPR) and the Data Protection Act 2018).
4. I understand that my information may be subject to review by responsible individuals from Addis Ababa University or King’s College London (UK) for monitoring and audit purposes.
5. I understand that confidentiality and anonymity will be maintained, and it will not be possible to identify me in any research outputs.
6. I agree to be contacted in the future by Addis Ababa University or King’s College London researchers who would like to invite me to participate in follow up studies to this project, or in future studies of a similar nature.
7. I agree that the research team may use my data for future research and understand that any such use of identifiable data would be reviewed and approved by a research ethics committee. (In such cases, as with this project, data would/would not be identifiable in any report).
8. I understand that the information I have submitted will be published as a report.
9. I agree that the information that I provide can be used for educational or research purposes, including publication and data archiving. In these publications and archives my confidentiality and anonymity will be maintained and it will not be possible to identify me or my child.
10. I consent to brief audio-visual recordings being made of my child.

1. I agree that the information I provide can be shared with a third party such as a university or a research institution for research purposes.

**__________________ __________________ _________________**

**Name of Participant Date Signature**

**Where participant/parent/guardian cannot read, a witness* may observe consent process and sign below if needed:**

Witness Statement (in event that participant is not literate):

I ­­­­­­­­­­­­______________________________________________ agree that the research project named above has been explained to ____________________________ (participant) to his/her satisfaction and that s/he agrees to take part in the study. Both the notes written above and the Information Sheet about the project have been read to him/her and his/her questions are answered properly.

**_________________ _________________**

**Date Signature**

**[Following section is recommended, and where verbal consent is obtained, must be signed by person undertaking informed consent.]**

I have followed the study procedure to obtain consent from the [participant]. S/he apparently understood the nature and the purpose of the study and consents to their participation in the study. S/he has been given opportunity to ask questions which have been answered satisfactorily.

Designee/investigator’s signature: ____________________________ Date ____________

Designee/investigator’s name: _____________________________Time ____________

(Please print name)

1. **Sample information sheet for caregivers to participate in randomised controlled trial of the CST (NB Ethiopian participants receive the form below in Amharic translation; Kenyan participants receive a version in Kiswahili, with all forms referring to Kenyan institutions and institutional review boards)**


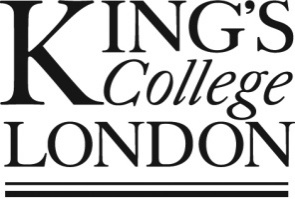
 **
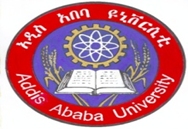
**

**Study Title: SuPporting African communities to increase the Resilience and mental health of Kids with developmental disabilities and their caregivers (SPARK).**

| **Institution** | **Investigators** |
| --- | --- |
| Addis Ababa University | Prof Charlotte Hanlon, Prof Abebaw Fekadu, Dr Fikirte Girma, Dr Girmay Medhin, Dr Tigist Zerihun, Dr Rehana Abdurahman, Dr Ruth Tsigebrhan, Dr Tsegereda Kifle |
| King's College London | Led by Dr Rosa Hoekstra |
| Aga Khan University, Institute of Human Development and KEMRI Wellcome Trust Research Programme | Led by Prof Amina Abubakar and Prof Charles Newton |
| University of Oxford | Prof Charles Newton |
| World Health Organization | Dr Chiara Servili |

You are being asked to take part in a study. The box below tells you important things you should think about before deciding to join the study. We will provide more detailed information below the box. Please ask questions about any of the information before you decide to participate. You may also wish to talk to others (for example, your family, friends, or your doctor) about this study, before agreeing to join.

| **Key Information for You to Consider** |
| --- |
| - **Voluntary Consent**. You are being asked to volunteer for a research study regarding developmental disabilities in children aged between 2 and 9 years. You can choose whether you would like to participate or not. If you do agree you can change your mind at any time and withdraw from the research. This will not affect you/your child’s care now or in the future. - **Purpose**. The World Health Organization developed a training package for caregivers of children with developmental disabilities (DD). The training aims to help caregivers to better understand the challenges their child may face and to teach caregivers skills to support their child’s development. The training was developed by an international group of experts. In a separate study in Ethiopia, our team found the training to be feasible and acceptable. In this study, we want to evaluate how well the training programme works in improving wellbeing and mental health of Ethiopian children with developmental disabilities and their caregivers. - **Risks or disadvantages.** A disadvantage for you is that participation in the programme will take time: around 2-3 hours each week to attend the group sessions, a further hour and a half during each of the three home visits, and up to an hour and a half each time you meet with the data collector. Moreover, it will take some time to practice what you learned during the training at home with your child. You may also feel upset, anxious, or distressed while participating in this programme. - In this study, participants will be “randomized” into one of two study groups. “Randomized” means that you are put into a group by chance, like a lottery. You will have an equal chance of being placed in either group. One of the two groups (intervention group) will be invited to receive the training straight away and the second group called wait-list group will wait for around ten months to receive the same intervention until participants in the intervention group have completed the training with the re-assessment of the outcome measures.   If you are allocated to the wait group a disadvantage for you is that you have to wait ten months before you can begin with the programme. However, a possible advantage of being allocated to this group is that you will not be asked to give extensive feedback on the programme, so will not have to spend much time on this. The research team has no influence on the group to which you will be allocated, this will be done randomly, like a lottery.  We recognise that you may find it distressing at times to discuss your child’s difficulties. The aim of the programme is to provide you with support, both from the facilitators who lead the programme sessions, as well as mutual support provided by other caregivers, who may experience similar difficulties. However, should you wish to withdraw from the programme then you are free to do so at any time.  **Benefits.** We hope that taking part in the training programme will be helpful to you. The programme was developed to support caregivers like you, to equip you with confidence and skills to support your child and help you to better understand your child. During the study period, we will cover the cost for registering or renewing your community-based health insurance. |

**What will happen if I agree to take part?**

If you decide to take part, you will be given this information sheet to keep and will be asked to sign a consent form. You will either be invited to take part in the programme straight away or will be asked to wait around ten months before starting with the training programme. Once your training programme starts, you will be invited to attend nine training sessions at a health centre. The sessions will be led by local facilitators (trained specifically to lead this programme) and will be attended by a group of caregivers who all have a child with developmental disabilities. The sessions will be held weekly, and each session will last approximately 2-3 hours. During these group sessions you will learn more about the condition of your child. There is no cure for your child’s condition. But in this training, you will learn skills to help you support your child in their communication, learning and development and to address behavioural challenges. The sessions also provide an opportunity to exchange experiences with other caregivers if you wish to do so. You are asked to come to each session by yourself, without your child if possible. You will be reimbursed for the costs of travelling to the health centre. Throughout the training you will be given exercises to try out at home with your child. In order to get the most out of the training you will be asked to spend some time during the week to try out these exercises with your child.

In addition to the group sessions, one of the trainers will visit you and your child three times: once before the first group session, once before or after the fifth group session, and once after the final group session. It is probably easiest for these visits to take place in your home, but we can also agree on another location convenient to you. The first home visit is made so that the trainer has a good understanding of the main challenges you and your child are facing and your needs in order to set training goals specific to your family’s needs. The second and third home visit are intended to evaluate progress and to identify any additional needs and challenges. We may also ask your permission to make a video recording of the trainer and your child while they are doing an activity together during the home visit. The purpose of this video recording is so supervisors who are supporting the trainer can help them get better at their job training you. However, if you are not agreeable to the video being recorded then you are free to say so, this will not affect your eligibility to take part in the programme.

**What will happen if I am allocated to the group that starts the training programme straight away?**

Throughout the programme we would also like to ask you more detailed questions about yourself, your child, and your experiences with the programme. A data collector will meet you at a location and time convenient to you three times during the programme: once before the programme starts, once immediately after the last session and once about six months after the last session. These interviews are conducted by an independent data collector (not one of the programme facilitators), so that you can speak freely about your thoughts of the programme. There are no right or wrong answers to any of the questions asked.

**What will happen if I am allocated to the group that waits a few months before starting the training programme?**

If you are allocated to the group that is to wait a few months before starting the training programme, a data collector will meet you at a location and time convenient to you three times during these months: once soon after signing up for this project, and then again four months after the initial assessment and ten months after the initial assessment. The data collector will ask you questions about you and your child. There are no right or wrong answers to any of the questions asked. After the third assessment is completed, you will be invited to take part in the training programme, consisting of nine group sessions and three home visits, as described in more detail above.

**Who will have access to the information I give?**

- All information we collect is stored securely in these locations, (1) locked university cabinets, (2) on encrypted password-protected computers used by our research team, (3) on a REDCap database hosted by Addis Ababa University, and (4) on King’s College London’s SharePoint storage platform. All electronic data resulting from the project will be stored on a secure computer network, and all data will be anonymised so that the interview and test results cannot be directly linked to contact details. On the written material we will use a code number instead of your name so that only the research team will know that this information comes from you. The video tapes will include footage of your child. We will make sure these data are kept confidential, by ensuring that the video tape data is encrypted, and password protected, and does not include any further personal information from you (e.g., your name or your child’s name or where you live). The code and your name will be kept in a separate, password-protected file. We will ensure that the report describing this study does not identify your individual contribution or your child. In order to do this study, we will share anonymised individual and summary information we collect or generate with investigators at Addis Ababa University, with Aga Khan University and the Kenya Medical Research Institute (KEMRI) in Kenya in ways that do not reveal individual participants’ identities.
- In future, information collected or generated during this study may be used to support new research by other researchers in Ethiopia and other countries on identification of children with developmental disabilities. In all cases, we will only share information with other researchers in ways that do not reveal individual participants’ identities. For example, we will remove information that could identify people, such as their names and where they live, and replace this information with number codes.

**Data Protection Statement**

Your data will be processed under the terms of UK data protection law (including the UK General Data Protection Regulation (UK GDPR) and the Data Protection Act 2018). If you would like more information about how your data will be processed in accordance with GDPR please ask us for a separate information sheet that provides information on this topic.

Any video recording of your child will be kept securely stored and will be destroyed within two years of completion of the study. Your contact information and the consent form will be kept (securely stored) for ten years after the study has finished. The information from the assessments (but without giving the identity of you or your child) will be archived and kept for a minimum of ten years after the study is completed.

Your rights to access, change or move your information are limited, as we need to manage your information in specific ways in order for the research to be reliable and accurate. You can withdraw from the study until 10 months after this consent date. Because you contribute to a study that is testing an intervention, we cannot fully remove your individual contributions from all systems. However, we can remove all your personal information (so you can no longer be identified or contacted by the research team) and can ensure your data is removed from all analyses. You can find out more about how we use your information by contacting the study investigators (details below).

**Who has allowed this research to take place?**

All research at Addis Ababa University has to be approved before it begins by a national and international committee who look carefully at planned work. They must agree that the research is important, relevant to Ethiopia and follows nationally and internationally agreed research guidelines. This includes ensuring that all participants’ safety and rights are respected. In Ethiopia this project has been approved by Addis Ababa University College of Health Sciences Institutional Review Board. Internationally, this research has been approved by the Health Faculties Research Ethics Subcommittee (RESC) at King's College London (UK).

**How is the study being funded?**

This research is funded by the National Institute of Health and Care Research in the UK.

**What will happen if I refuse to participate?**

All participation in research is voluntary. You are free to decide if you want to take part or not. If you do agree you can change your mind at any time without any consequences.

**What if I have further questions?**

You are free to ask us any question about this research. If you have any further questions about the study, you are free to contact the research team using the details below for further advice and information:

Please contact Dr XX on YYY or email ZZZ or Dr XXX on YYY or email YYY or Dr Rosa Hoekstra at XX.

**If you want to ask someone independent anything about this research, please contact:**

Institutional Review Board of the College of Health Sciences of Addis Ababa University, email address XX and phone number YY

**What if something goes wrong?**

If this project has harmed you in any way or if you wish to make a complaint about the conduct of the project you can contact:

(In Ethiopia) Institutional Review Board of the College of Health Sciences of Addis Ababa University, email address XX and phone number YY

(In London) The Chair, Health Faculties Research Ethics Subcommittee (RESC) at King's College London (UK) at XX.

If you wish to raise a complaint on how we have handled your personal data, you can contact our Data Protection Officer, Mr XX at YY who will investigate the matter.

**Thank you for reading this information sheet and for considering taking part in this research.**

*THE PARTICIPANT SHOULD NOW BE GIVEN A SIGNED COPY TO KEEP*
